# Bibliometric analysis of focal therapy in prostate cancer research

**DOI:** 10.1002/bco2.353

**Published:** 2024-03-17

**Authors:** Mohammed Shahait, Sarah Ibrahim, Laith Baqain, Zahi Abdul‐Sater

**Affiliations:** ^1^ Department of Surgery Clemenceau Medical Center Dubai United Arab Emirates; ^2^ Global Health Institute American University of Beirut Beirut Lebanon; ^3^ Faculty of Medicine University of Jordan Amman Jordan; ^4^ College of Public Health Phoenicia University Mazraat El Daoudiyeh Lebanon

**Keywords:** bibliometric, focal therapy, prostate cancer

## Abstract

**Introduction:**

The use of focal therapies for prostate cancer (PCa) has soared, as it controls disease and is associated with minimal side effects. Bibliometric analysis examines the global research landscape on any topic to identify gaps in the research and areas for improvement and prioritize future research efforts. This study aims to examine the research outputs and trends and collaboration landscape in the field of focal therapy for PCa on a global scale.

**Methods:**

We searched Medline, PubMed and Scopus for peer‐reviewed publications on our research topic using controlled keywords. Search results were limited to the period between 1980 and 2022, screened for duplicates and then included in our study based on prespecified eligibility criteria. The Bibliometrix Package was used for comprehensive science mapping analysis of co‐authorship, co‐citation and co‐occurrence analysis of countries, institutions, authors, references and keywords in this field.

**Results:**

This analysis included 2578 research articles. The annual scientific production increased from one article in 1982 to 143 in 2022 (13.21%). The average citation per year was incrementally increasing, and these documents were cited around 32.52 times. The documents included in this analysis were published in 633 sources. The international collaboration index was 22.7. In total, 6280 author keywords were identified. The most used keywords were ‘prostate cancer’, ‘focal therapy’, ‘prostate’ and ‘photodynamic therapy’.

**Conclusion:**

This bibliometric analysis has provided a comprehensive review of focal therapy in PCa research, highlighting both the significant growth in the field and the existing gaps that require further exploration. The study points to the need for more diverse international collaboration and exploration of various treatment modalities within the context of focal therapy.

## 1 INTRODUCTION

Prostate cancer (PCa), one of the most prevalent malignancies globally, stands as the third leading cause of cancer‐related deaths among men in the United States of America (USA).^1^, ^2^ While surgery and radiation have been the traditional treatment of PCa, a growing body of evidence suggests that overdiagnosis is prevalent and that these traditional approaches may not alter the natural history of the disease or improve the quality of life for patients.^3^, ^4^ In light of this, the United States Preventive Services Task Force issued a statement advising against performing prostate‐specific antigen (PSA) screening, attributing it to the risks of overdiagnosis and overtreatment of low‐risk prostate PCa.^4^ Consequently, prominent urology associations have underscored the importance of active surveillance (AS) in managing low‐risk PCa, positioning it as the preferred initial management strategy not only for low‐risk PCa but also for a select group presenting with favourable intermediate‐risk PCa.^3^ Despite the acknowledged role of AS in managing PCa, the urology community continues to grapple with determining the optimal candidates for this approach. The criteria for AS, follow‐up regimens and intervention triggers differ across institutions, further complicating this decision.^5^ Additionally, AS can escalate anxiety among some patients, stemming from perceived inaction and potential loss to follow‐up.^6^

Offering a middle ground between AS and radical treatments for PCa with a low risk of metastasis, focal therapies such as targeted laser therapy, high intensity focal therapy (FT) and electroporation are an emerging treatment option that seek to target only the cancerous tissue in the prostate, while preserving healthy tissue and minimizing the risk of side effects.^7^ Research into focal therapies for PCa aims to identify ideal candidates for treatment, improve patient outcomes and discover new treatment modalities, as the field of FT for PCa is evolving rapidly.^7^ The use of focal therapies for PCa has gained significant attention in recent years due to its potential to effectively treat localized PCa while minimizing side effects.

However, despite the increasing interest in FT, to our knowledge, no comprehensive bibliometric analysis has examined the global research landscape on this topic. Without this understanding, it is challenging to identify gaps in the research, areas for improvement and prioritize future research efforts in FT for PCa. This study aims to examine the research outputs and trends, and collaboration landscape in the field of FT for PCa on a global scale.

## 2 METHODS

### 2.1 Source of data

A bibliometric review was conducted to understand the research landscape of focal therapies for PCas, globally. Bibliometrics is a statistical analysis and quantitative tool to evaluate the growth, impact and trend of scientific publications. The Web of Science (WoS) was the source of the bibliographic data, as it contains the most comprehensive information about scientific research and is the most used in bibliometric analysis.^8^

### 2.2 Search strategy

A comprehensive search of all publications related to focal therapies for prostate cancer publication was carried from 1980 to 2022. A search strategy was developed by compiling an extensive list of PCas and focal therapies‐related keywords, which were identified from previous studies, reviews, meta‐analyses, among others (Supporting Information S1). Boolean operators (AND, OR and NOT) were used to conduct the search. The search was further refined to include human studies only by excluding keywords related to ‘Canine’ from all titles and subject ‘veterinary sciences’ from the search strategy.

### 2.3 Inclusion criteria

Publications related to benign cancers, canines or veterinary sciences were excluded. The period of the index date was 1 January 1980 to 31 December 2022. The document type included was only original research articles, with the language restricted to English. All other types of documents, including review articles, case reports, editorials, books and letters, were excluded.

### 2.4 Data management and selection process

#### 2.4.1 Data extraction and analysis

The final list of references was then analysed. The analysis was completed using the Bibliometrix Package, which is an R statistical software package for comprehensive science mapping analysis.^9^ The raw data exported from R were transformed into graphics and tabular format using the Flourish software to generate the following information: (a) annual scientific production and article citation; (b) journals in which researchers publish; (c) country‐specific production; (d) author's countries; (e) collaboration patterns; and (g) author's keywords and title co‐occurrence.

### 2.5 Ethical approval

This study is exempt from the American University of Beirut Institutional Review Board (IRB) since the researchers used publicly available information and did not involve any interactions with human participants.

## 3 RESULTS

### 3.1 Mapping the characteristics of the publications

**FIGURE 1:**
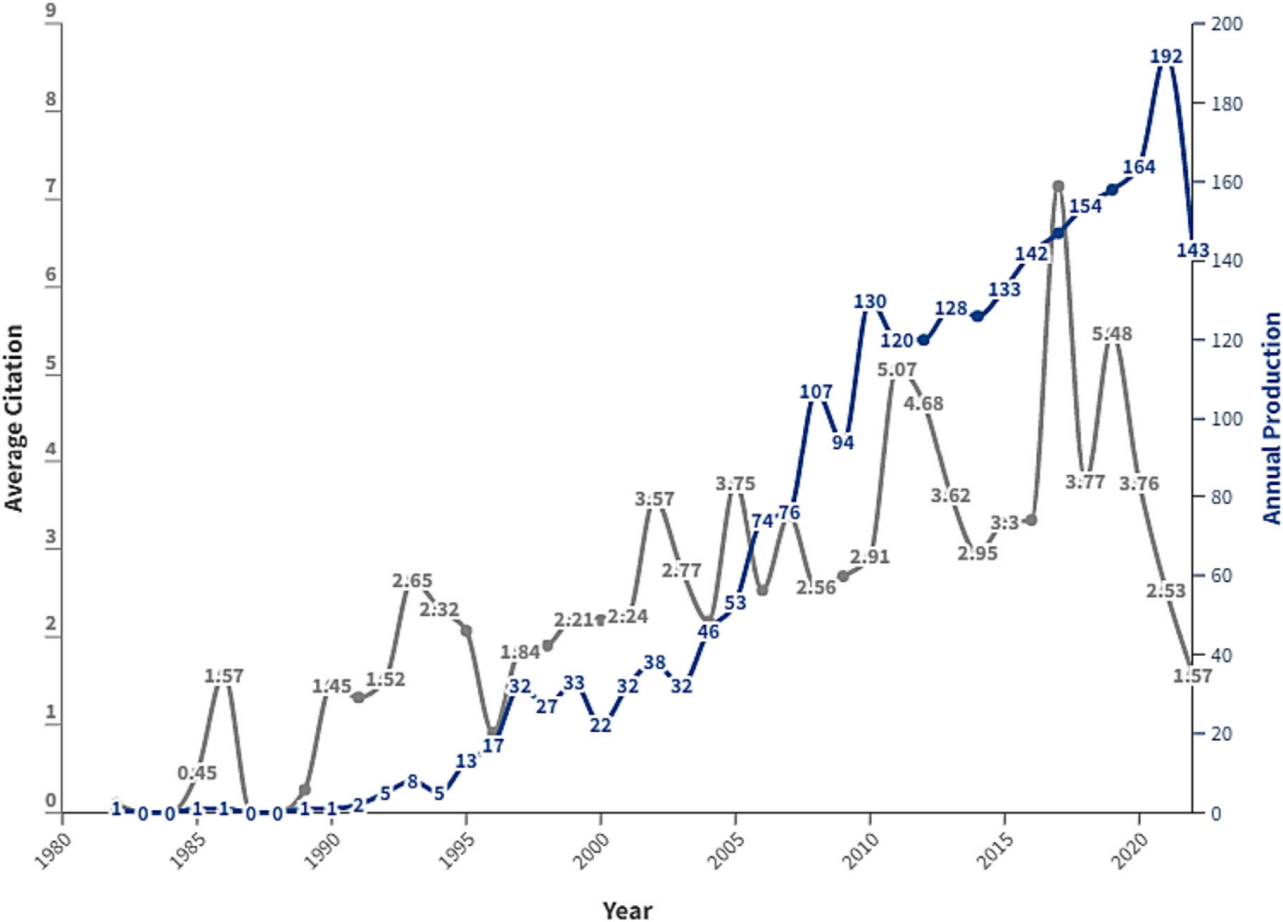
Annual scientific production and article citation per year on focal therapies for prostate cancer research from 1980 to 2022

The annual number of documents published in the last four decades is depicted in Figure 1. The annual scientific production increased from one article in 1982 to 143 in 2022 (13.21%) (Figure 1). Despite that, the research output was mainly driven by authors from a limited number of countries. The average citation per year was incrementally increasing, with notable spike in numbers of citations post 2010. On average, these documents were cited around 32.52 times (Figure 1).

### 3.2 Analysis of source

**FIGURE 2:**
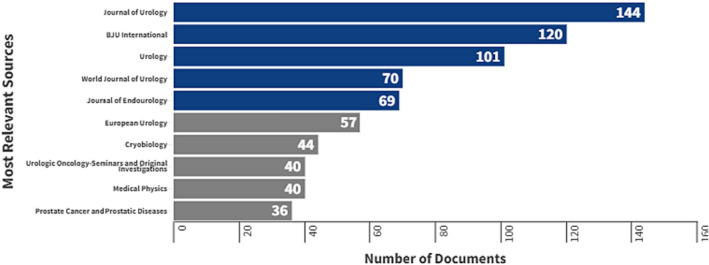
Journals with 35 or more publications on focal therapies for prostate cancer research from 1980 to 2022

The documents included in this analysis were published in 633 sources including journals (Table 1). Around 14% of the articles were published in the Journal of Urology (impact factor [IF] 6.6), British Journal of Urology International (IF 5.9) and Urology (IF 2.6). Figure 2 presents the journals with 35 or more articles.

**TABLE 1:** Summary of the main information of collected bibliometric data on focal therapies for prostate cancer research from 1980 to 2022

Description	Results
Documents	2578
Sources (journals, books, etc.)	633
Keywords plus (ID)	4195
Author's keywords (DE)	6280
Period	1982–2022
Average citation per document	32.52
Authors	10 611
Authors of single‐authored documents	50
Authors per documents	7.06
Collaboration Index	22.7

### 3.3 Analysis of countries

**FIGURE 3:**
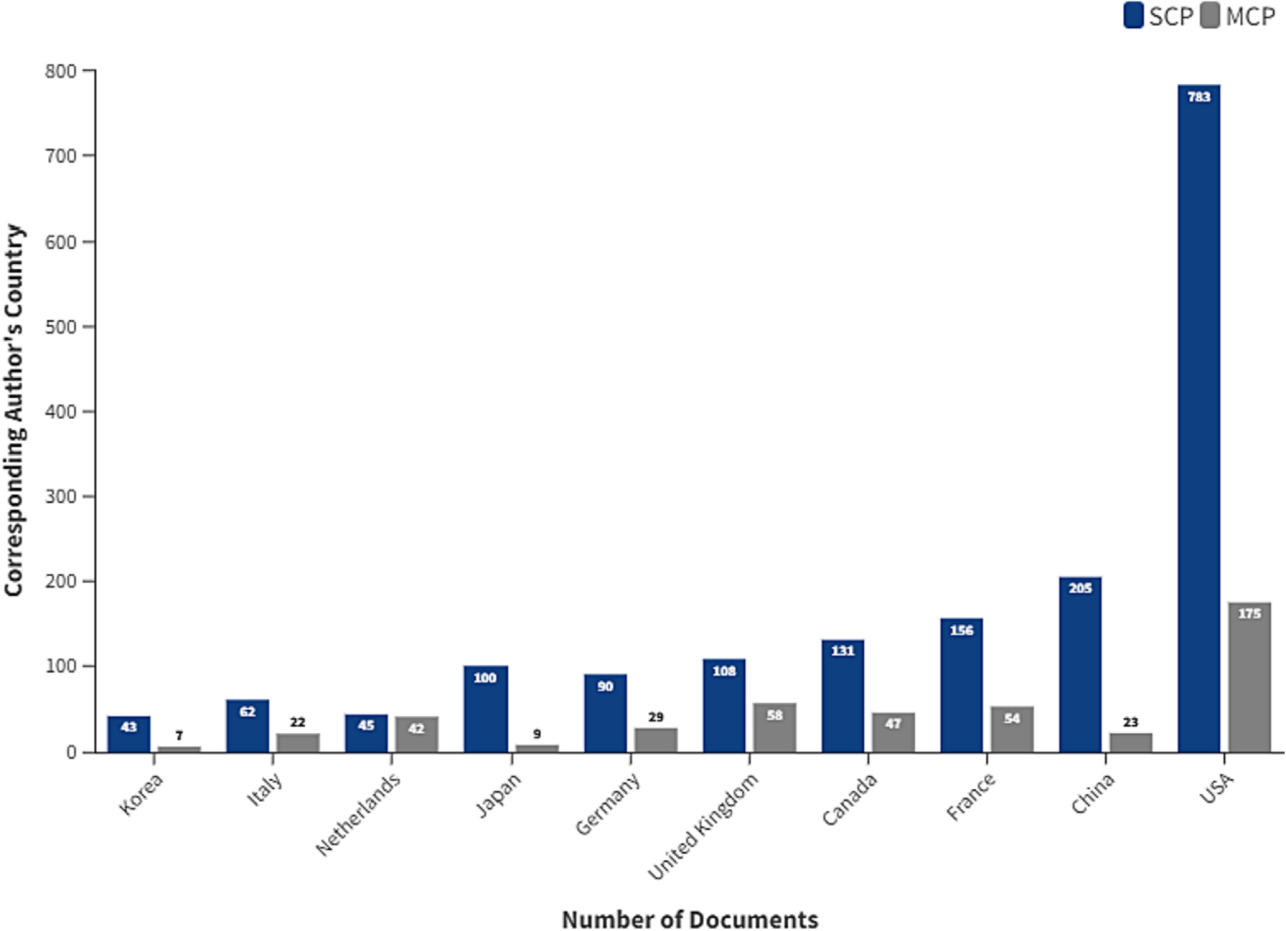
Top 10 corresponding authors' countries on focal therapies for prostate cancer research from 1980 to 2022. The dark blue bars demonstrate the publication rate by authors from the same country (Single Country Publication; SCP). The grey bars represent the number of publications by the corresponding author's country, with at least one foreign co‐author existing (multiple countries publication, MCP)

When the data were analysed by country, the highest number of corresponding authors for single country publication was from the USA (763), followed by China (205), France (156), Canada (131) and the United Kingdom (108) (Figure 3). While the corresponding authors for multiple country publication were hailed mainly from the USA (175), the United Kingdom (56), France (54) and Canada (47). The number of coauthors per document was 7.06 per document (Table 1). The international collaboration index was 22.7 (Table 1).

**FIGURE 4:**
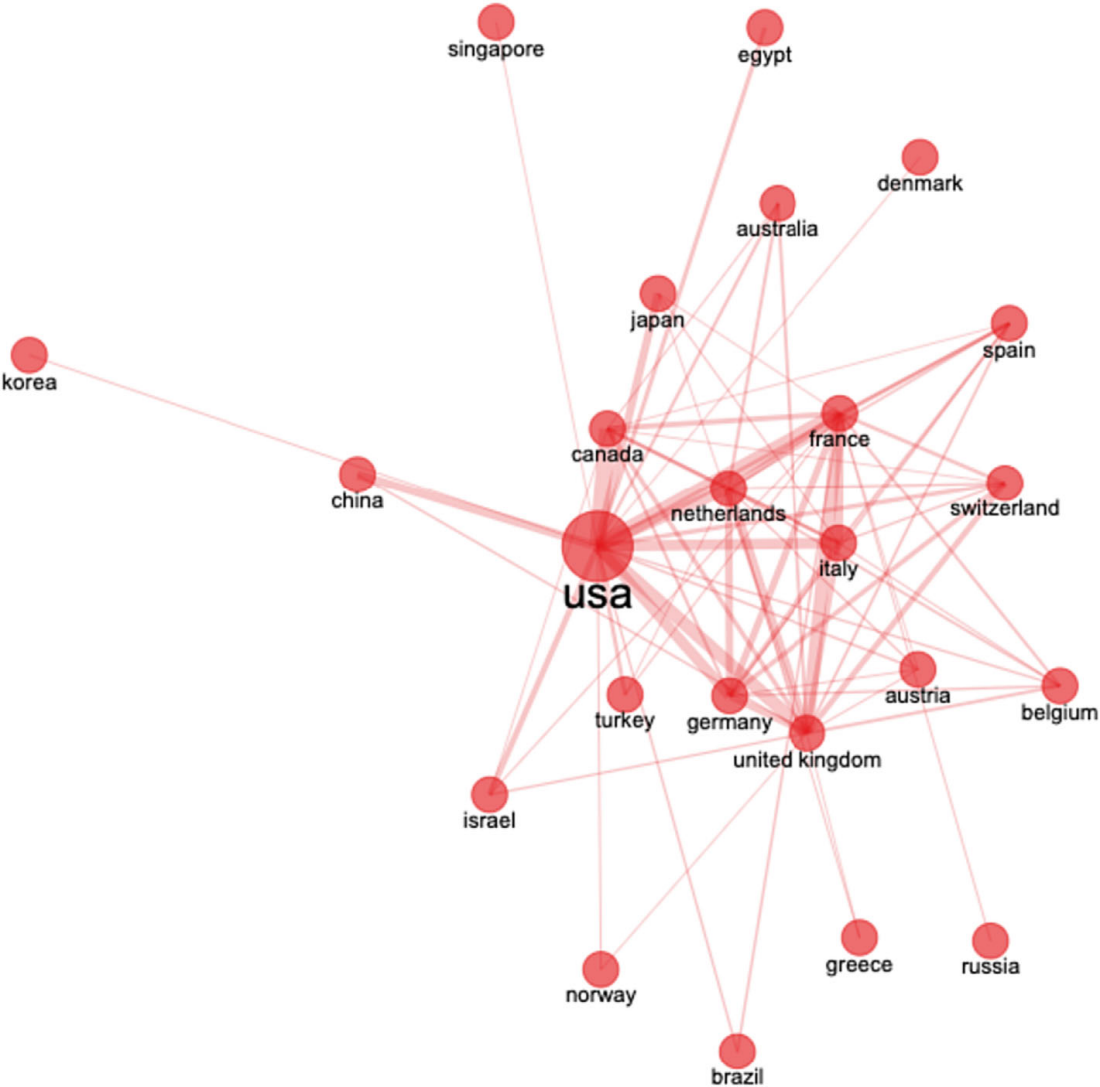
Country collaboration on focal therapies for prostate cancer research from 1980 to 2022. The font size and circle diameter indicate the number of publications in the country within the dataset. The lines' thickness demonstrates the strength of the collaboration between the countries.

Multiple collaborations have taken place among researcher around the globe on the topic of FT of PCa; however, our analysis sheds light on a strong collaboration between the USA and the United Kingdom, France, Canada, Netherland and Italy (Figure 4).

### 3.4 Author's keywords and title co‐occurrence

**FIGURE 5:**
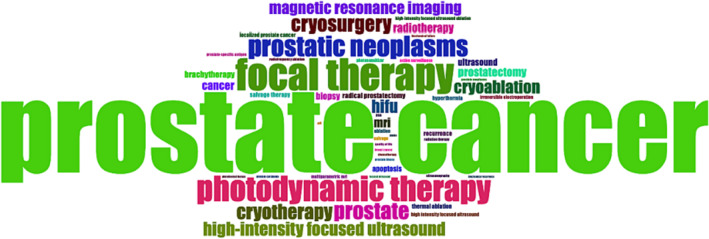
Word cloud for top author keywords focal therapies for prostate cancer research from 1980 to 2022

**FIGURE 6:**
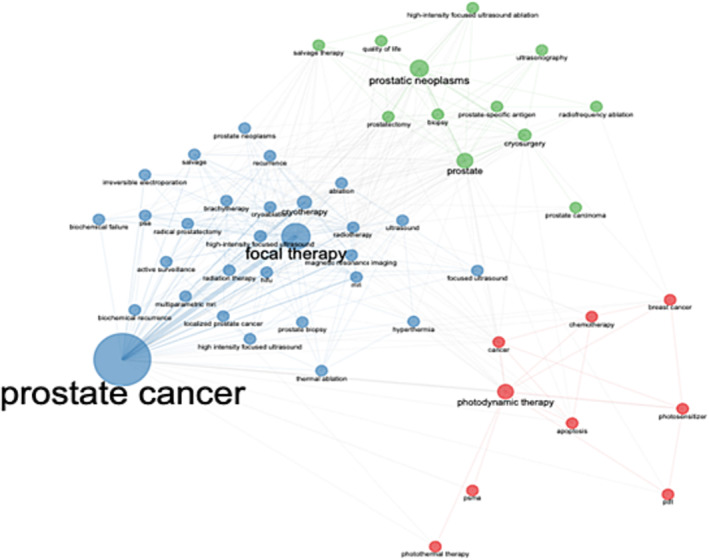
Co‐occurrence by authors' keywords on focal therapies for prostate cancer research from 1980 to 2022. The circle diameter represents the occurrence of the keyword. The edge size is proportional to item co‐occurrence. Co‐occurring keywords are grouped under different colours.

To further characterize the research on FT in PCa, the author's keywords and title co‐occurrence were analysed. The bibliometric data on the FT in PCa research identified 6280 author keywords (Table 1). A world cloud was used to summarize keywords and their occurrences. The most used keywords were ‘prostate cancer’, ‘focal therapy’, ‘prostate’ and ‘photodynamic therapy’ (Figure 5). The co‐occurrence of title used in FT in PCa research displayed two major themes (Figure 6).

## 4 DISCUSSION

This study is the first to summarize the current research status of focal therapies in PCa through a bibliometric analysis and report on the publication source, collaborations and keywords utilization. The query of the main biomedical bibliographic databases for peer reviewed publications pertaining focal therapies in PCa in the last 40 years revealed 2578 unique articles, published in 633 different sources.

In the past four decades, different forms of FT for various cancers and benign conditions have been developed. As such, the application of focal therapies in PCa has exponentially increased in the last decade after USPSTF statement against PCa screening, and this is evident by the increase in research output after 2012. Institutions and authors based in the USA are leading the efforts in advancing focal therapies research in PCa as they collectively published more than 750 articles in the field. Authors and institutions from other countries, such as China, Germany, France and the United Kingdom, are following the footsteps of their colleagues and institutions based in the USA.

Our study yielded an overall increase in research output pertaining to FT, from one article to 143 between the years 1982 to 2022 as shown in Figure 1. The increase in scientific production is very likely related to the more fund allocations to support this field, while it would have been desirable to witness a more diverse representation and contribution from different regions worldwide. Our findings showed all countries—except Italy and Israel—with the most contributions pertaining to FT (Figure 6) appear to be among the top 25 leading countries in oncology research overall.^8^

The USA with the most productivity in research is responsible for 32.4% (of all publications), followed by the United Kingdom (9.4% of all publications). The top five countries (USA, the United Kingdom, France, Canada and China) are responsible for 66.2% of the total research output.

According to our analysis, there has been a substantial surge in research studies focusing on FT in PCa over the past 25 years, demonstrating a significant fivefold increase. This significant rise aligns with the ongoing upward trend observed in the broader field of oncology research.^8^

The volume of articles found in these journals points to the cancer‐related scientific community's strong inclination for publishing their research in these particular outlets. The Journal of Urology had the most with 144 articles pertaining to FT (5.6% of the total), followed by BJU international and Urology (4.7% and 3.9%, respectively).

In addition, there has been an overall increase in citations over the last two decades as shown in Figure 2. Citations evaluate the growth in input and serve as a valuable tool for assessing the enduring impact of an article, quantified through the frequency of its references in scholarly works. In the academic context, the productivity and success of authors are frequently associated with their capacity to generate articles that bring in significant citation counts.^9^, ^10^ When addressing individual performances, Agostinis P,2011 CA‐Cancer Journal led with 3286 total citations. We must address that the average citations may be affected by the time since an article's publication, potentially leading to an underestimation of the true value of recently published articles.

In addition to volume, research quality holds equal significance when evaluating the research output.^11^ Therefore, in this study, we analysed the development of article quality and scientific impact using the average citations per paper per year from 1982 to 2022 as a predictive parameter. It has been demonstrated that the average citations per year parameter is indicative of the number of authors contributing to the field.^11^

Establishing and strengthening the existing international collaborations can also help in improving the quality by expanding the possibilities for innovation and diversifying research expertise and resources.^12^ Multiple collaborations have taken place among researchers around the globe on the topic of FT of PCa; however, as shown by Figure 3, our analysis sheds light on a strong collaboration between the USA and the United Kingdom, France, Canada, Netherlands and Italy. The network analysis emphasizes the significant role of the USA, evident by its largest node, indicating a higher number of research partnerships compared to other countries. We could appreciate similar results seen in a study done by Chadegani et al.,^8^ where the USA, France and the United Kingdom had the most prominent collaborators.

As shown in Figure 6, when the data were analysed by country, the highest number of corresponding authors for single country publication was from the USA (763), followed by China (205), France (156), Canada (131) and the United Kingdom (108), while the corresponding authors for multiple country publication were hailed mainly from the USA (175), the United Kingdom (56), France (54) and Canada (47). Australia was the only country among the highest contributors, to have more multiple country publications than single country publication. Moreover, the institutions with the most research output on FT of PCa were University of Toronto with 180 published articles, followed by Memorial Sloan Kettering cancer centre with 125 articles.

In the context of urology‐oncology publications, the steady increase in the output across the years can be owed to the remarkable increase in funding.^13^ Funding has long been established as an important factor that influences the quantity, impact and citations of research articles.^14^, ^15^ While Nassereldine et al.^16^ have shown in the MENA region, only 21.5% of the publications received financial support, indicating a relatively low level of funding for research in this area. Insufficient funding for medical research continues to be a significant obstacle in the MENA region, with only a small fraction of MENA‐based centres contributing to cancer research funding compared to the global average.^17^, ^18^, ^19^

Despite rising FT research, substantial gaps persist in energy type, FT candidates and follow‐up. Further international collaboration can bridge these gaps, improving PCa FT. Collaborations enable data sharing, facilitating comprehensive analyses for complex questions. Moreover, explore FT impact across populations, addressing treatment disparities among different regions and communities. As still PCa poses a challenge due to current cancer care system limitations and the rising disease burden.^20^ The strong collaborations identified between specific countries, such as the USA, the United Kingdom, France, Canada, Netherlands and Italy, open up opportunities for innovation and diversified research expertise. However, more international collaboration is needed to address gaps in literature, especially concerning the type of energy for FT, candidates for FT and follow‐up schemes.

### 4.1 Limitations

For our analysis, we conducted a bibliometric study and relied on the data provided by the databases we selected. These databases served as our primary source of information for the study. An incomplete portrayal of the overall research landscape may be due to unintentionally exclusion of non‐peer‐reviewed publications. Additionally, the reliance of researchers on indexing selected databases creates potential bias. Different databases include different ranges of journals and publications, impacting the comprehensiveness of the analysis. Search strategies must also be exercised with caution as selection of keywords, search terms and filters can influence the outcomes and potentially exclude relevant publications. Moreover, bibliometric analyses tend to heavily rely on publications in English, which can introduce a bias towards research conducted in English‐speaking countries. As a result, studies conducted in other languages and regions may be underrepresented in the analysis. Lastly, due to extensive time to collect and analyse data, time lag between publication and research outputs may limit inclusion of these studies in our finding.

Future directions should focus on enhancing international collaboration, exploring diverse populations and addressing health disparities in access to treatments. Further research into energy types, candidate selection and follow‐up schemes for FT may lead to improved outcomes for PCa patients. Investment in research quality and value, along with efforts to overcome limitations such as language and regional biases, will be essential as the field continues to advance our understanding and management of FT for PCa.

## 5 CONCLUSION

This bibliometric analysis has provided a comprehensive review of FT in PCa research, highlighting both the significant growth in the field and the existing gaps that require further exploration. The study points to the need for more diverse international collaboration and exploration of various treatment modalities within the context of FT. As PCa continues to pose a significant global challenge, it is imperative that future research addresses the identified gaps, including the selection of energy types for FT, candidate identification and follow‐up protocols. Investment in research quality, inclusivity of diverse regions and overcoming language biases will be essential in advancing the field. We hope that the results of this study will serve as a roadmap for researchers, guiding the direction of future work to improve outcomes for PCa patients and contribute to the ongoing evolution of FT as an effective treatment option.

## AUTHOR CONTRIBUTIONS

Mohammed Shahait contributed to the design implementation of the research and to the writing of the manuscript and Sarah Ibrahim and Laith Baqain to the analysis of the results and to the writing of the manuscript. Zahi Abdul Sater conceived the original and supervised the project.

## CONFLICT OF INTEREST STATEMENT

The authors declare that they have no affiliations with or involvement in any organization or entity with any financial interest in the subject matter or materials discussed in this manuscript.

## Data Availability

Data are available upon request from the reviewer.
